# An Injectable, Dual Responsive, and Self-Healing Hydrogel Based on Oxidized Sodium Alginate and Hydrazide-Modified Poly(ethyleneglycol)

**DOI:** 10.3390/molecules23030546

**Published:** 2018-03-01

**Authors:** Lei Wang, Wanfu Zhou, Qingguo Wang, Chao Xu, Quan Tang, Haiyang Yang

**Affiliations:** 1 CAS Key Laboratory of Soft Matter Chemistry, School of Chemistry and Materials Science, University of Science and Technology of China, Hefei 230026, China; wl1991@mail.ustc.edu.cn (L.W.); xc94@mail.ustc.edu.cn (C.X.); tangq@mail.ustc.edu.cn (Q.T.); 2 Oil Production Technology Institute, Daqing Oilfield Company Ltd., Daqing 163453, China; zhouwf@petrochina.com.cn (W.Z.); wqg@petrochina.com.cn (Q.W.)

**Keywords:** oxidized sodium alginate, dynamic covalent bonds, stimuli-responsive, sol-gel transitions, injectable hydrogel, self-healing

## Abstract

Oxidized sodium alginate is a handily modifiable polysaccharide owing to the pendant aldehyde groups which can form dynamic covalent bonds with amines, acylhydrazines, etc., providing oxidized sodium alginate-based hydrogels with stimuli-responsive properties. However, due to the stiffness and, in particular, the hydrophobicity of sodium alginate dialdehyde at low pH, the mechanical performance and pH stimuli responsiveness of oxidized sodium alginate-based hydrogels are still strictly limited. Herein, we report a new strategy to build an injectable, dual responsive, and self-healing hydrogel based on oxidized sodium alginate and hydrazide-modified poly(ethyleneglycol) (PEG). The hydrazide-modified PEG, referred to as PEG-DTP, acts as a macromolecule crosslinker. We found that the presence of PEG-DTP reduces the hydrophobicity of oxidized sodium alginate at low pH so effectively that even a pH-induced reversible sol-gel transitions can be realized. Meanwhile, the disulfide bonds in PEG-DTP endows the hydrogel with the other reversible sol-gel transitions by redox stimuli. In particular, due to the softness of PEG-DTP chains, mechanical performance was also enhanced significantly. Our results indicate we can easily integrate multi-stimuli responsiveness, injectability, and self-healing behavior together into an oxidized sodium alginate-based hydrogel merely by mixing an oxidized sodium alginate solution with PEG-DTP solution in certain proportions.

## 1. Introduction

With high water content and their structural similarity to natural extracellular matrix (ECM), hydrogels have be widely studied and applied in the fields of drug delivery [1], tissue engineering [2], and wound healing [3]. To serve as biomaterials, especially as the carriers [4] of drugs or cells scaffold [5], hydrogels should incorporate the properties of biocompatibility, injectability, and self-healing. Meanwhile, for better practical applications, some recently developed hydrogels are endowed with attractive external stimuli-responsiveness. The injectability facilitates the hydrogel to incorporate therapeutic drugs and cells via simple mixing and a minimally invasive [6] surgical procedure, and with a decreased risk of implant migration [7]. Self-healing hydrogels are soft materials which can quickly repair the damaged network. In case of unexpected breakage of the gel matrix and the subsequent inflammation risk caused by the fragmented gels, the ability to self-heal is important for hydrogels applied in vivo [8]. Besides, some dynamic covalent interactions or physical interactions are utilized to prepare hydrogels with stimuli-responsive properties [9,10,11]. By the triggers of pH [12,13], reducing agents [14], temperature [15], or light [16], hydrogels can be degraded and the incorporated drugs can be released. For example, Zhang et al. [17] prepared multi-responsive polymer hydrogels based on the dynamic boronate ester and disulfide bonds. In response to pH, glucose, and redox, the hydrogel exhibited gel–sol-gel transitions, showing the potential use as drug carrier. Therefore, if the hydrogel is simultaneously injectable, self-healing [18,19,20], and stimuli-responsive, it would be of great value.

As a natural polymer, sodium alginate is biocompatible and in an oxidizing environment part of its constitutional units can be oxidized to aldehyde groups through ring opening reactions [21]. The sodium alginate dialdehyde (ADA) with numerous aldehyde groups can react with a number of groups, such as amines, to form dynamic covalent bonds [22]. The sodium alginate-based hydrogels, therefore, can be endowed easily with stimuli-responsive properties. As an important crosslinker, 3,3′-dithiobis(propionohydrazide) (DTP) [23] with hydrazide and disulfide functional groups can react with aldehyde groups by Schiff base reactions to from acylhydrazone crosslinks. Since the Schiff base reaction can be quickly conducted at mild conditions, hydrogels are able to form in situ after mixing and injecting the precursor solutions. For example, injectable pH-responsive hollow particle gels utilizing DTP via Schiff base reaction were prepared by Brian R. Saunders [24]. Besides, the dynamic acylhydrazone bonds and disulfide linkages endow the hydrogels with self-healing and stimuli-responsive properties. However, due to the stiffness of the oxidized sodium alginate chain, the hydrogels based on oxidized sodium alginate crosslinked by DTP are brittle. In particular, the oxidized sodium alginate itself will dehydrate in acidic conditions, showing a poor pH responsiveness.

Herein, we synthesized sodium alginate dialdehyde (ADA) and 3,3′-dithiobis (propionohydrazide) (DTP) modified PEG (PEG-DTP) to prepare an injectable, stimuli-responsive, and self-healing hydrogel. PEG-DTP/ADA hydrogel was prepared via the Schiff base reaction between PEG-DTP and ADA. The soft macromolecular crosslinker (PEG-DTP) improved the flexibility of the network effectively and the mechanical performance of PEG-DTP hydrogel was obviously improved. Besides, the more hydrophilic crosslinker PEG-DTP obviously enhanced the hydrophilicity of the hydrogel network, contributing to a better pH responsiveness of the hydrogel. Since the gelation time can be tuned by adjusting pH, the PEG-DTP/ADA hydrogel showed good injectability. The dynamic acylhydrazone bonds which can self-regenerate after breaking provide the hydrogel with a 100% self-healing ability at room temperature. Moreover, the pH-sensitive acylhydrazone bonds and redox-sensitive disulfide linkages endowed the PEG-DTP/ADA hydrogel with dual responsive properties. Accordingly, the degradation of the hydrogel was controllable by varying pH or reductant concentrations. The hydrogel was applied to control release of rhodamine B and distinct 1,4-dithiothreitol (DTT) controlled release behaviors were observed. Because of the outstanding biocompatibility of sodium alginate and PEG, the PEG-DTP/ADA hydrogel was non-cytotoxic, as testified by in vitro cytotoxicity evaluation.

## 2. Results and Discussion

### 2.1. Preparation and Rheological Characterization of PEG-DTP/ADA Hydrogel

Aldehyde sodium alginate (ADA) was the oxidative product of sodium alginate, oxidized by sodium periodate (Figure S1). The oxidization of ALG to ADA was verified by the FT-IR spectra of sodium alginate (ALG) and oxidized sodium alginate (ADA) (Figure S2), Due to the absorption peak of ADA, a new absorption peak corresponding to the characteristic band of a carbonyl group of ADA appeared at 1726 cm^−1^, showing the successful preparation of ADA. The oxidation degree of sodium alginate was about 35%, as determined by the hydroxylamine hydrochloride titration method [25]. PEG-DTP was obtained by the amidation between DTP and PEG-diacid (Figure S3). The structures of PEG-diacid and PEG-DTP were characterized by ^1^H-NMR. As shown in Figure S3, the peak b (4.45 ppm) on the spectrum of PEG-diacid originated from the methene near the carboxy group, which demonstrated that poly(ethyleneglycol) was oxidized to PEG-diacid. In Figure S4, peak “c” (2.88 ppm, methene near the disulfide bond) and “d” (2.51 ppm, methene near the hydrazide bond) on the ^1^H-NMR spectrum indicated that PEG-diacid and DTP had reacted to form PEG-DTP.

**Figure 1:**
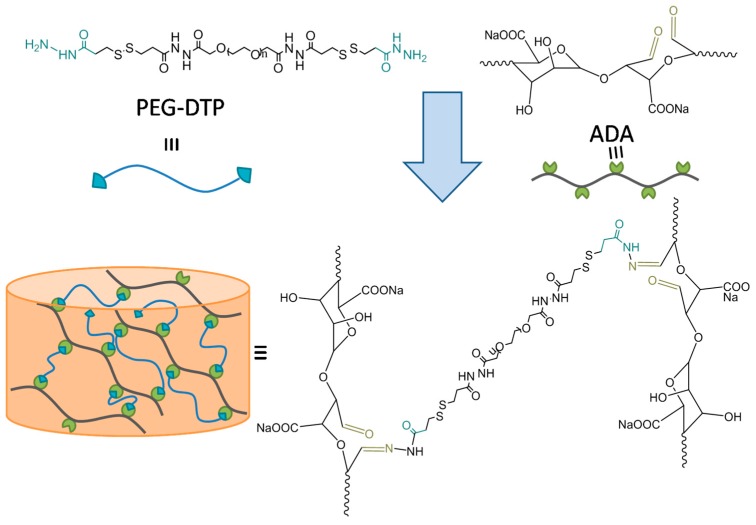
Schematic diagram of the preparation of the PEG-DTP/ADA hydrogel.

As we all know, The Schiff base reaction can be conducted at mild conditions (room temperature, wide pH range) to form hydrogel. As depicted in Figure 1, the hydrogel was facilely prepared by mixing the aqueous ADA solution (20 wt %) with the aqueous PEG-DTP solution (20 wt %). The Schiff base reaction between ADA and PEG-DTP is quite fast, such that the dynamic network was quickly formed and the gel was obtained in several minutes (Figure S5). A series of PEG-DTP/ADA hydrogels with different mass ratios of PEG-DTP/ADA and different solid content were prepared, noted as Gel_X_-Y (Table S1). Herein, “Y” means the mass ratio of two components (PEG-DTP:ADA = 1, 2, 3, 4) and “x” means different solid content of hydrogels (15 wt % and 20 wt %).

### 2.2. Mechanical Properties of ADA/PEG-DTP Hydrogels

**Figure 2:**
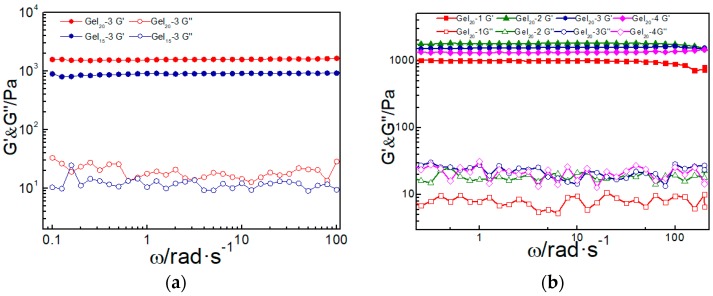
(**a**) Frequency sweep measurements of hydrogels Gel_15_-3 and Gel_20_-3; (**b**) Storage moduli (G′) and loss moduli (G″) of hydrogels Gel_20_-1, Gel_20_-2, Gel_20_-3, and Gel_20_-4 as a function of angular frequency.

The mechanical properties of the prepared hydrogels were measured by rheometer (TA Instruments, AR-G2). As shown in Figure 2a, the elastic modulus G′ of the hydrogel was larger than the loss modulus G″ in the whole frequency range, demonstrating the sample was always in the gel state. As solid content increased, the elastic modulus G′ of the Gel_20_-3 hydrogel was higher than the Gel_15_-3 hydrogel, although they had similar loss modulus G″ (20 Pa). The better elasticity of Gel_20_-3 indicated that more robust crosslinking networks were formed in the high solid content hydrogel, resulting in better mechanical performance. When varying the mass ratio of the two components of the hydrogels (PEG-DTP:ADA = 1, 2, 3, 4), the G′ of Gel_20_-3 was higher than other hydrogels, while the G″ of these hydrogels were similar (Figure 2b). This revealed that the mass ratio of PEG-DTP:ADA = 3:1 was optimum to form a strong ADA/PEG-DTP hydrogel. With better mechanical properties, Gel_20_-3 was selected to test the other properties of the ADA/PEG-DTP hydrogels, as displayed below.

**Figure 3:**
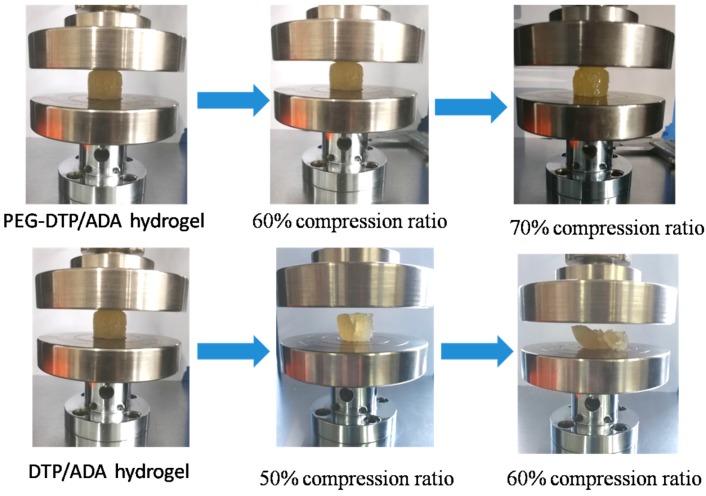
Compression performance of the PEG-DTP/ADA hydrogel and the DTP/ADA hydrogel.

It is noted that the PEG-DTP/ADA hydrogel showed better mechanical properties than the DTP/ADA hydrogel crosslinked by DTP (Figure 3). The PEG-DTP/ADA hydrogel was able to sustain a compressive strain of over 60%, whereas the DTP/ADA hydrogel was broken at a compressive strain of 50%. In the DTP/ADA hydrogel, simply crosslinking the stiff ADA molecule with the small molecule crosslinker DTP generated a quite rigid network and the resulting hydrogel was brittle. By contrast, the soft macromolecular crosslinker PEG-DTP could effectively improve the flexibility of the network. Thus, the mechanical performance of the PEG-DTP hydrogel was obviously improved.

### 2.3. Self-Healing and Injectability of PEG-DTP/ADA Hydrogels

**Figure 4:**
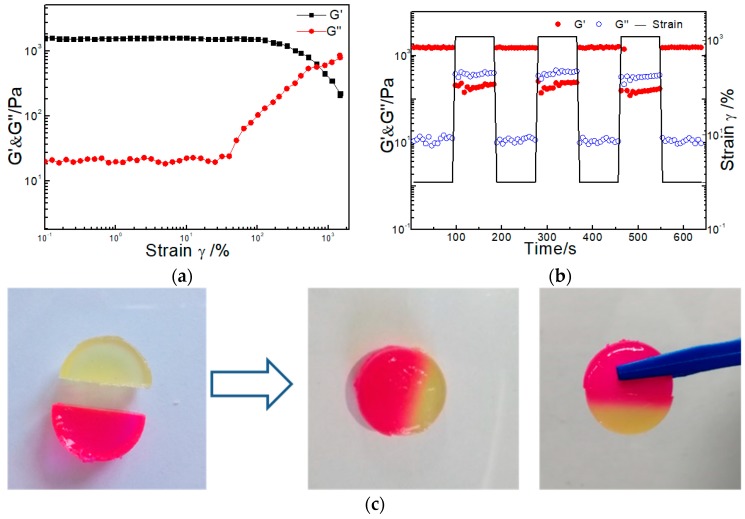
(**a**) Strain sweep measurements of hydrogel Gel_20_-3; (**b**) Repeated dynamic strain step tests (1% or 1200%); (**c**) Images of the self-healing process of two individual gel fragments.

As mentioned before, to quickly repair a damaged gel matrix, the ability to self-heal is important for the practical application of hydrogels, especially for injectable hydrogels. Before testing the self-healing ability, we conducted a strain sweep measurement on Gel_20_-3 to test the maximum shear strain the hydrogel could withstand. As shown in Figure 4a, the storage moduli G′ and the loss moduli G″ of the hydrogel did not change under the strain γ < 60%, showing that the sample could bear a large shear strain. With the strain increased, G′ dropped while G″ dramatically raised and the curve had a crossover at strain γ = 700%. This indicated the network of hydrogel was totally broken and converted into sol state at strain γ = 700%. Then, in order to test the self-healing ability of the hydrogel, repeated dynamic strain step tests (γ = 1% or γ = 1200%) were carried out (Figure 4b). When the strain increased to 1200%, the hydrogel network was disrupted as the storage moduli G′ was only 200 Pa and the loss moduli G″ increased to 400 Pa. After decreasing the strain back to 1%, the G′ and G″ values of the sample almost recovered to initial values, implying the hydrogel network was able to completely restore itself after it was broken. The tests were repeated three times, and in each cycle, G′ and G″ of the sample could recover their initial values, further illustrating the 100% and repeatable self-healing functionality of the PEG-DTP/ADA hydrogels. Besides, Figure 4c also visually demonstrated that the hydrogels had a good self-healing ability at room temperature. The hydrogels were dyed with different colors and cut into two halves. When put them together, the two parts self-healed into an integral hydrogel after 12 h. 

**Figure 5:**
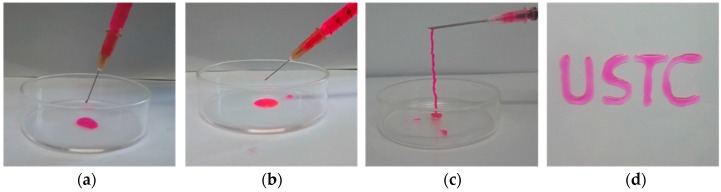
Injection of ADA solution (**a**) and PEG-DTP solution (**b**), injectability (**c**), and printing ability (**d**) of hydrogel samples. (Both compositions were dyed by rhodamine B).

Since the Schiff base reaction between PEG-DTP and ADA can quickly establish chemical crosslinks to form the hydrogel network (Figure 1), the injectability of the PEG-DTP/ADA hydrogel was shown in Figure 5. ADA solution and PEG-DTP solution were in the liquid state when squeezed out (Figure 5a,b). The ADA solution and the PEG-DTP solution were mixed together in a syringe and the gel was formed in situ when squeezed out. For better injectability, the gelation time of the injectable hydrogel should be controllable. The gelation time of the ADA/PEG-DTP hydrogels can be easily controlled due to the pH sensitivity of the Schiff base reaction. As depicted in Table S2, gelation time measured by vial inversion test [26] at different pH values (3.0, 5.0, 7.0) were 10 s, 60 s, and 1.5 h. Namely, the gelation time of the hydrogel can be tuned over a wide range by changing the pH. Figure 5d shows that the PEG-DTP/ADA hydrogels can be squeezed as bio-ink to write the word “USTC”. Together with the tunable gelation time, the PEG-DTP/ADA hydrogel is also a promising candidate as a 3D printing hydrogel.

### 2.4. Dual Responsive Properties of PEG-DTP/ADA Hydrogels

**Figure 6:**
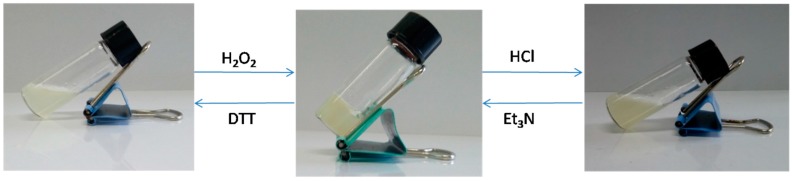
Sol-gel transitions of PEG-DTP/ADA hydrogels in response to pH or redox triggers.

The reaction between the –NH_2_ groups of PEG-DTP and the –CHO groups of ADA formed the pH-sensitive acylhydrazone bonds, and the disulfide linkage in PEG-DTP were responsive to reductants (DTT or GSH). Thus, the dual responsive properties were displayed in our PEG-DTP/ADA hydrogel system. Acylhydrazone bonds could break at low pH and reform at high pH, while disulfide linkages could break in reducing environments and reform in oxidizing environments, thus the hydrogel exhibited dual sol-gel transitions. Vial inversion tests [26] were used to monitor the gel–sol-gel transition. As shown in Figure 6, the hydrogel turned into the sol-gel state by adding hydrochloric acid solution to turn the pH to 1. Then, when triethylamine was added to change the pH to 7, the hydrogel was reformed again. Furthermore, the sol-gel transition was also observed by the triggering of redox stimuli (DTT/H_2_O_2_). The hydrogel turned into the sol-gel state when adding the reductant, DTT, and recovered the hydrogel network after adding the oxidant, H_2_O_2_ (Figure 6). The dual sol-gel transitions indicated that the PEG-DTP/ADA hydrogel has excellent stimuli-responsiveness.

**Figure 7:**
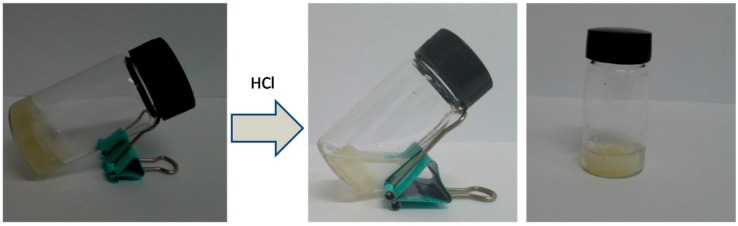
Dehydration of DTP/ADA hydrogel at pH = 1.

In contrast, the DTP/ADA hydrogel did not show the pH triggered sol-gel transition (Figure 7). The hydrogel was dehydrated in acidic solutions, which was attributed to the decreased hydrophilicity of the hydrogel network resulting from the protonation of carboxyl groups in the ADA segments. The replacement by DTP in the more hydrophilic crosslinker PEG-DTP obviously improved the hydrophilicity and flexibility of the hydrogel network, contributing to the outstanding pH responsiveness of the hydrogel.

### 2.5. Degradation and Drug Release Ability of PEG-DTP/ADA Hydrogels

**Figure 8:**
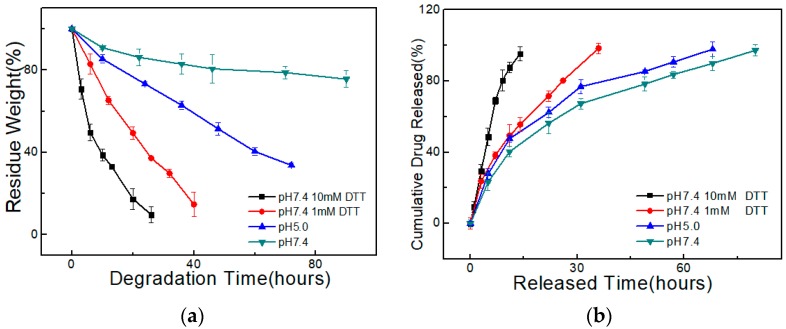
(**a**) Degradation and (**b**) release of a model drug (rhodamine B) by the hydrogels.

For injectable hydrogels, controllable degradability is of high value for drug release. Due to the stimuli responsiveness, the PEG-DTP/ADA hydrogel exhibited intriguing pH/redox triggered degradation. Reductants, such as DTT and glutathione (GSH), could break the disulfide crosslinking and hasten the degradation of the hydrogel network. As shown in Figure 8a, the weight loss of the PEG-DTP/ADA hydrogel (Gel_20_-3) was only 20 wt % in 3 days in pH 7.4 PBS solution. While immersed in DTT solutions for 26 h, the hydrogel showed almost 90% weight loss in 10 mM DTT solution and 63% weight loss in 1 mM DTT solution. The obviously different and accelerated degradation rate verified that the hydrogel’s degradation ratio can be easily controlled by varying the concentration of DTT. Besides, since acylhydrazone bonds were cleavable under acidic conditions, the degradation of PEG-DTP/ADA hydrogel could be triggered by varying pH. As shown in Figure 8a, the hydrogel showed a higher degradation rate at pH = 5, compared to pH = 7.4. Therefore, the degradability of the ADA/PEG-DTP hydrogel can be easily controlled by adjusting the DTT concentration or pH.

With the controllable degradation of the hydrogel network, the drug release rate of a PEG-DTP/ADA hydrogel can be easily controlled. To study the drug release ability of the hydrogel, we used rhodamine B as the model drug. The in vitro drug release behavior tests were carried out in weakly acidic and/or reductive conditions. As shown in Figure 8b, rhodamine B released nearly 100% in 12 h in 10 mM DTT solution, while it took nearly 40 h to release 100% in 1 mM DTT solution. On the contrary, when in the pH 7.4 PBS solution, it took 80 h to completely release the drug. Similarly, in pH 5.0 solution, the rhodamine B released more quickly than in pH 7.4 solution. In view of the biologically relevant microenvironments such as tumor sites, the controllable release of drug under weakly acidic and reductive conditions makes the hydrogel a promising drug carrier.

### 2.6. In Vitro Cytotoxicity Evaluation

**Figure 9:**
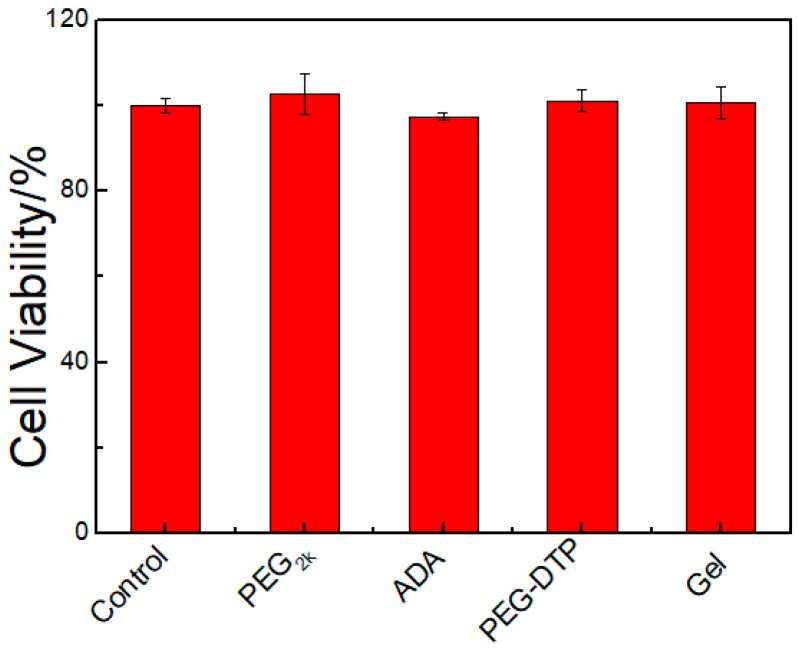
Cell (A549) viability upon exposure to PEG_2k_, ADA, PEG-DPT, and hydrogel.

As it is well known that poly(ethyleneglycol) and sodium alginate are biocompatible polymers and are widely used in hydrogel preparation, the PEG-DTP/ADA hydrogels are supposed to be biocompatible. A549 cells were utilized to study the cell cytotoxicity of the hydrogel, and were analyzed by MTT assay [27]. The polymer solutions (PEG, PEG-DTP, and ADA) and hydrogel were used to test cell cytotoxicity. As shown in Figure 9, the A549 cells of PEG-DTP solution and ADA solution showed good viability (>90%) after incubation with the solution for 24 h. The results indicated that PEG, PEG-DTP, ADA, and hydrogel had good biocompatibility, confirming that the hydrogel is a good candidate for use as a drug carrier.

## 3. Materials and Methods

### 3.1. Materials

1,4-Dithiothreitol (DTT), dicyclohexylcarbodiimide (DCC), and *N*-hydroxysuccinimide (NHS) were purchased from Aladdin Reagents (Shanghai, China). 3,3′-Dithiobis (propionohydrazide) (DTP) was purchased from J&K Scientific (Shanghai, China). Sodium alginate (ALG), sodium periodate, methanol, ethanol, ethylene glycol, poly(ethyleneglycol) (PEG, Mn = 2000), phenolphthalein, concentrated HCl solution, dimethyl sulfoxide (DMSO), triethylamine (Et_3_N), and sodium hydroxide were obtained from Sinopharm Reagents (Shanghai, China) and were used as received without further purification. Murine A549 cells were purchased from SIBCB (Shanghai Institute of Cell Biology, Shanghai, China). Cell culture lysis buffer and MTT salt (3-(4,5-dimethylthiazol-2-yl)-2,5-diphenyltetrazolium bromide) and FBS (fetal bovine serum), trypsin, PBS (phosphate buffered saline), and DMEM (Dulbecco’s modified Eagle medium) were purchased from GIBCO (Beyotime Institute of Biotechnology, Shanghai, China).

### 3.2. Synthesis of Sodium Alginate Dialdehyde (ADA)

Oxidized sodium alginate (ADA) was synthesized according to previously reported literature [28]. The specific steps are as follows (Figure S1). First, 10 g sodium alginate (ALG) was dissolved in 500 mL deionized water in 1 L round bottom flask, and then 5.50 g sodium periodate was dissolved in 100 mL deionized water. The two solutions were mixed together and stirred for another 6 h in a dark environment at room temperature. Ethylene glycol, equimolar to sodium periodate, was added and stirred for an hour to quench the reaction. The product mixture was precipitated with excess ethanol. Then, the oxidized product of sodium alginate (ADA) was filtrated and dialyzed (MWCO = 8000~10,000) for two days. Finally, pure ADA was obtained by freeze-drying. 

### 3.3. Synthesis of Hydrazide-Terminated Poly(ethyleneglycol) (PEG-DTP)

3,3′-Dithiobis(propionohydrazide) (DTP) functionalized poly(ethyleneglycol) (PEG-DTP) (Figure S1) was prepared by the amidation reaction [10] between PEG-diacid and 3,3′-dithiobis (propionohydrazide). First, PEG-diacid was synthesized according to a similar procedure of the previous report [29]. Twenty grams of PEG_2k_ was dissolved into 200 mL acetone in a 500 mL round bottom flask. Then, the solution was heated to 40 °C to obtain a clear solution and then it was cooled to room temperature. Next, 10 mL Jone’s reagent (1.25 M) was added dropwise and the reaction mixture was kept at room temperature. The reaction mixture was stirred overnight (24 h). Then, the reaction was quenched by 6.5 mL (equimolar to Jone’s reagent) isopropyl alcohol. After evaporating off the acetone, the crude product was dissolved by 150 mL deionized water. The product was extract by dichloromethane (30 mL × 3). Finally, PEG-diacid was obtained after solvent evaporation.

To prepare PEG-DTP, 8 g 3,3′-dithiobis(propionohydrazide) (DTP), 1.16 g DCC, and 0.65 g NHS were dissolved into 80 mL DMSO in a 250 mL round bottom flask. Then, 10 g PEG-diacid dissolved into 20 mL DMSO was added dropwise over 1 hour, and stirring of the reaction mixture was continued overnight (24 h). After evaporating off DMSO, the product mixture was collected and dialyzed (MWCO = 1000) over 2 days. Finally, the PEG-DTP obtained by freeze-drying.

### 3.4. Preparation of PEG-DTP/ADA Hydrogel

PEG-DTP/ADA hydrogel was prepared by a simple procedure. The details are as follows. PEG-DTP and ADA were dissolved in PBS solution (pH = 7.4). Then, 0.25 g ADA aqueous solutions (20 wt %) and 0.75 g PEG-DTP aqueous solutions (20 wt %) were mixed at room temperature. Several minutes later, the mixed solutions gelled. The obtained 20 wt % hydrogel was named as Gel_20_-3. Using Gel_20_-3 (Table S1) as the sample, the experiments of injectability, sol-gel transition, in vitro cytotoxicity evaluation, and self-healing were carried out. Detailed components of other gels are presented in Table S1. 0.25 g ADA aqueous solution (20 wt %) and 0.75 g DTP aqueous solutions (15 mg/mL) were mixed to form DTP/ADA hydrogel. 

### 3.5. Characterizations

A Nicolet IS-10 spectrometer (Thermo Fisher, Waltham, MA, USA) was used to record the FT-IR spectra of ADA and ALG. The scan interval was 4000–600 cm^−1^, with 32 scans at a resolution of 2 cm^−1^.

A rheometer (TA Instruments, AR-G2, New Castle, DE, USA) with a platform of 4 cm diameter was used to measure hydrogels’ rheological behavior. The storage moduli G′ and loss moduli G″ were studied at a constant-strain (1%) mode. The frequency range was 0.1–100 rad s^−1^. The self-healing properties of hydrogels were measured at a constant frequency of 6.283 rad s^−1^ by a strain step cycled between 1% and 1200% at 25 °C.

The gel–sol-gel transition experiment was conducted by inversion. HCl (5 M) and triethylamine were used to trigger pH responsive gel–sol-gel transitions. First, HCl (10 μL, 5 M) was added to the hydrogel. After 12 h, equimolar triethylamine was added. For redox responsive gel–sol-gel transitions, we used DTT and H_2_O_2_ as the trigger. First, 16 mg DTT was added into the 1 g hydrogel samples. After 12 h, equivalent H_2_O_2_ (~10 μL) was added and the hydrogels reformed.

The degradation properties of PEG-DTP/ADA hydrogels were determined by retention in different solutions. Hydrogels were submerged in PBS buffer (pH = 7.4 and 5.0), 10 mM (pH = 7.4) DTT solution, and 1 mM DTT (pH = 7.4) solution. The hydrogels were freeze-dried and weighed (W_0_). Then, the hydrogels were immersed into PBS buffer (20 mL) or different concentrations of DTT solution (20 mL). After a certain period of time, the hydrogels were taken out, freeze dried, and weighed again (W_t_). W_t_/W_0_ × 100% was used to calculate the hydrogel’s degradation ratio. The tests were conducted three times to confirm precision.

Rhodamine B was used as the model drug to analyze the drug release ability of hydrogel. Several Gel_20_-3 hydrogels loaded with rhodamine B were prepared as follows. First, PEG-DTP solutions (0.75 g 20 wt %) and rhodamine B aqueous solution (10 μL, 1.0 mg/mL) were mixed to obtain a drug solution. Then, the drug solution and ADA solution (0.25 mL 20 wt %) were mixed to prepare drug-containing hydrogel samples. The four samples were incubated in 10 mL PBS (pH = 7.4, 5.0) buffer and 10 mL DTT (10 mM, pH = 7.4) solution or 10 mL DTT (1 mM, pH = 7.4) solution, respectively. At specific time points, sample solutions (4 mL) were withdrawn for UV-vis analysis and then the same volume of fresh solution was put back into the incubator. The 555 nm absorption of UV-vis were recorded to represent the concentration of rhodamine B. The tests were conducted three times. The cell compatibility of the designed hydrogels was studied by MTT assay. A549 cells cultured in 96-well plates at a density of 10^4^ cells per well were incubated at 37 °C under 5% CO_2_. One hundred microliters of DMEM with 10% fetal bovine serum and 5% penicillin were added to each well. After 24 h incubation, the DMEM was replaced with a fresh culture medium, and then the prepared polymer (PEG_2K_, ADA, and PEG-DTP) solutions (diluted to 1.5 wt %) and hydrogel extract were added at a specific concentration. Then, the cells were incubated for 24 h. The MTT solution (20 μL, 5 mg/mL in PBS buffer) was added to each well and then cultured for another 4 h. The medium in each well was removed and the purple formazan crystals from viable cells was dissolved by 200 μL DMSO. The cell viability was analyzed by the absorbance at a wavelength of 480 nm by a micro-plate reader (Thermo Fisher, Waltham, MA, USA). 

## 4. Conclusions

In this work, we used handily modifiable polysaccharide-oxidized sodium alginate and a macromolecular crosslinker, PEG-DTP, to prepare an injectable, dual responsive, and self-healing hydrogel via Schiff base crosslinking reaction. The stiffness and dehydration of oxidized sodium alginate at low pH caused the poor mechanical performance and pH responsiveness of the hydrogel. However, the introduction of PEG-DTP effectively improved the flexibility and hydrophilicity of hydrogel network and the ADA/PEG-DTP hydrogel exhibited better mechanical performance and a pH-induced reversible sol-gel transition. With the dynamic acylhydrazone bonds and disulfide linkages, the ADA/PEG-DTP hydrogel showed dual responsive (pH and redox) properties and the degradation rate or drug release of the hydrogel could be well controlled by varying the concentration of DTT solution or the pH. Moreover, the hydrogels could self-repair nearly 100% after breakage because of the reversible dynamic acylhydrazone bonds. The in vitro cytotoxicity tests showed the hydrogel had excellent cytocompatibility. Therefore, the sodium alginate-based hydrogel integrated with multi-stimuli responsiveness, injectability, and self-healing ability showed potential for use in the fields of drug delivery, tissue engineering, and controlled 3D cell culture, etc.

## Supplementary Materials

The following are available online. Figure S1: Schematic diagram of formation of ADA and PEG-DTP, Figure S2: FT-IR spectra of sodium alginate (ALG) and oxidized alginate (ADA), Figure S3: ^1^H-NMR of PEG-diacid in CDCl_3_, Figure S4: ^1^H-NMR of PEG-DTP in CDCl_3_, Figure S5: Photograph of PEG-DTP solution, ADA solution and hydrogel, Table S1: Preparation of PEG-DTP/ADA hydrogel with different contents, Table S2: Gelation time of Gel_20_-3 measured by vial inversion test.
