# Supplementary material for: An Injectable, Dual Responsive, and Self-Healing Hydrogel Based on Oxidized Sodium Alginate and Hydrazide-Modified Poly(ethyleneglycol)
Source: Molecules. 2018 Mar 1;23(3):546. doi: 10.3390/molecules23030546 (PMC6017758; doi:10.3390/molecules23030546)
Supplement: Supplementary file 1 [file molecules-23-00546-s001.pdf]

An Injectable, Dual Responsive and Self-Healing Hydrogel Based on sodium alginate dialdehyde and Hydrazide Modified Poly(ethyleneglycol)

Lei Wang [1], Wanfu Zhou [2], Qingguo Wang [2], Chao Xu [1], Quan Tang [1] and Haiyang Yang [1,*]

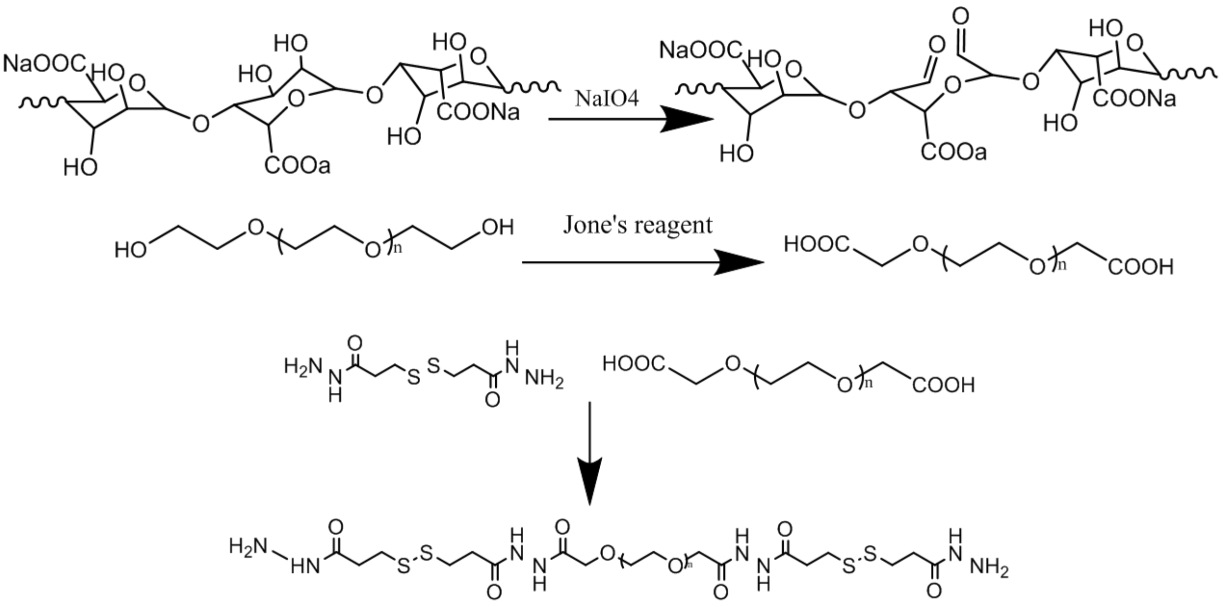

Figure S1. Schematic diagram of formation of ADA and PEG-DTP.

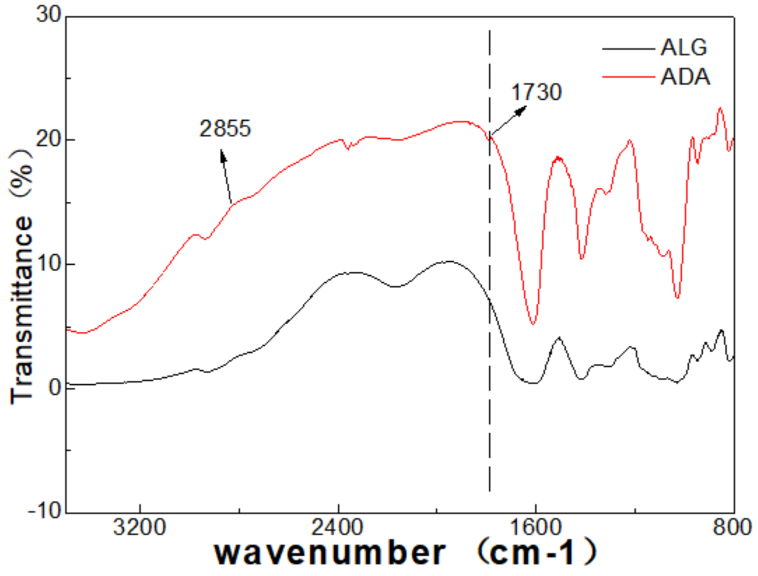

Figure S2. FT-IR spectra of sodium alginate (ALG) and oxidized alginate (ADA) performed by potassium bromide.

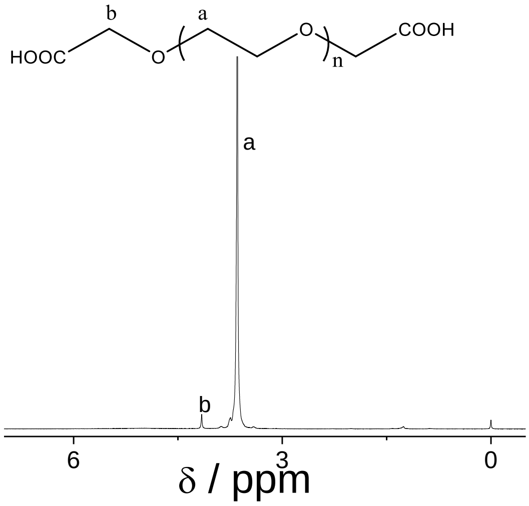

Figure S3. ^{1}H-NMR of PEG-diacid in $CDCl_3$.

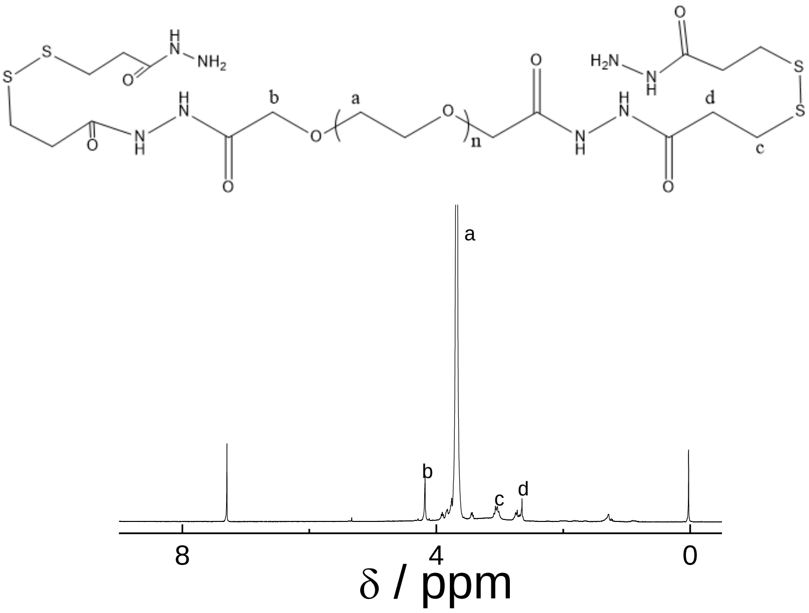

Figure S4. ^{1}H-NMR of PEG-DTP in $CDCl_3$.

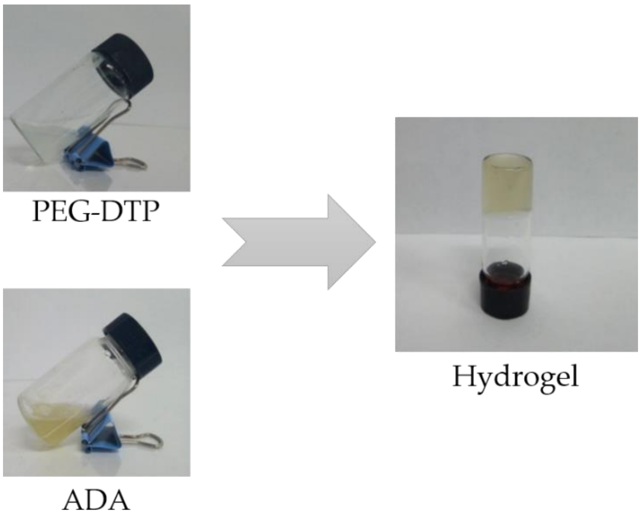

Figure S5. Photograph of PEG-DTP solution, ADA solution, and hydrogel

Table S1. Preparation of PEG-DTP/ADA hydrogel with different contents.

Mass ratio (ADA/PEG-DTP)	ADA solution （1g）	PEG-DTP solution （3g）	Sample name （wt%）
1/3	15wt%	15wt%	Gel_{15}-3(15wt%)
1/3	20wt%	20wt%	Gel_{20}-3(20wt%)

Mass ratio (ADA/PEG-DTP)	ADA solution （20wt%）	PEG-DTP solution （20wt%）	Sample name （wt%）
1/1	1g	1g	Gel_{20}-1 (20wt%)
1/2	1g	2g	Gel_{20}-2 (20wt%)
1/3	1g	3g	Gel_{20}-3 (20wt%)
1/4	1g	4g	Gel_{20}-4 (20wt%)

Table S 2. Gelation time of Gel_{20}-3 measured by vial inversion test.

pH	3	5	7
Time(s)	10	60	$5.4*10^3$
